# SARS-CoV-2 Virologic and Immunologic Correlates in Patients with Olfactory and Taste Disorders

**DOI:** 10.3390/microorganisms8071052

**Published:** 2020-07-15

**Authors:** Marco Benazzo, Irene Cassaniti, Eugenia Maiorano, Anna Calastri, Federica Novazzi, Alice Bonetti, Antonella Sarasini, Raffaele Bruno, Fausto Baldanti

**Affiliations:** 1 Department of Otorhinolaryngology, Fondazione IRCCS Policlinico San Matteo, 27100 Pavia, Italy; marco.benazzo@unipv.it (M.B.); eugenia_maiorano@libero.com (E.M.); anna.calastri90@gmail.com (A.C.); 2 Department of Clinical, Surgical, Diagnostic and Pediatric Sciences, University of Pavia, 27100 Pavia, Italy; raffaele.bruno@unipv.it; 3 Molecular Virology Unit, Fondazione IRCCS Policlinico San Matteo, 27100 Pavia, Italy; i.cassaniti@smatteo.pv.it (I.C.); federica.novazzi01@universitadipavia.it (F.N.); alice.bonetti01@universitadipavia.it (A.B.); a.sarasini@smatteo.pv.it (A.S.); 4 Infectious Diseases Unit, Fondazione IRCCS Policlinico San Matteo, 27100 Pavia, Italy

**Keywords:** anosmia, ageusia, SARS-CoV-2, serology

## Abstract

The main object of the study was to investigate the SARS-CoV-2 molecular and serological pattern in patients with mild symptoms including anosmia and ageusia. A cohort of 69 patients with olfactory and taste disorders (OTDs) were enrolled and prospectively monitored. Serological and molecular assays for the characterization of SARS-CoV-2 IgG and SARS-CoV-2 RNA, respectively, were performed at the time of enrolment and after 7 and 14 days. Patients were stratified according to the symptoms’ onset. A total of 52 patients (75.4%) were diagnosed as COVID-19 positive being SARS-CoV-2 RNA and/or SARS-CoV-2 IgG positive. The remaining 17 (24.6%) were negative for COVID-19 and excluded from the analysis. We reported that only 34 out of 52 patients (65.4%) were positive for SARS-CoV-2 RNA. Moreover, the median time from onset of symptoms and enrolment was significantly higher in those patients with negative SARS-CoV-2 RNA in nasal swabs, suggesting that symptoms might last longer than SARS-CoV-2 replication. The great majority of patients (80%) developed SARS-CoV-2 IgG at three weeks after symptoms’ onset while the detectability of SARS-CoV-2 RNA dramatically decreased over time, suggesting the crucial role of combination of molecular and serological assays for the diagnosis of COVID-19 in those patients reporting mild symptoms.

## 1. Introduction

Human coronaviruses (CoVs) are enveloped viruses with a single strand positive-sense RNA genome, belonging to the family Coronaviridae (subfamily Orthocoronavirinae) and divided into to four genus alpha-CoV (Group 1), beta-CoV (Group 2) within four lineages (A, B, C and D) are recognized, gamma-CoV (Group 3) and delta-CoV (Group 4). Strains HCoV-229E and HCoV-NL63 are included in alphacoronavirus genus, while HCoV-OC43, HCoV-HKU1, MERS and SARS are members of the betacoronavirus genus [1]. Despite that the largest part of human coronaviruses are mainly responsible for mild seasonal respiratory diseases, in 2003, a human coronavirus (SARS-CoV) caused the severe acute respiratory syndrome coronavirus (SARS) outbreak [2] and, in June 2012, 10 years after the first emergence of SARS-CoV, a novel coronavirus, Middle East respiratory syndrome coronavirus (MERS-CoV), was isolated in a man in Saudi Arabia died of acute pneumonia and renal failure [3].

In general, HCoVs come from animal reservoirs such as bats for NL63, SARS-CoV, and MERS while OC43 and HKU1 come from rodents [4]. Then, HCoVs spread from the animal host into the human population via an intermediate host species (cows, civets, camels, pangolins and minks) [4,5]. Consequently, the intra- and interspecies transmission of HCoVs is possible [1]. To date, no vaccines or specific therapies against HCoVs are available in order to prevent coronavirus infections; therefore, good hygienic practice and investigation of epidemiological features are necessary.

Recently, a SARS-related CoV was identified as the etiological agent responsible for an outbreak originating in Wuhan, central China. Indeed by December 2019 a new coronavirus named severe acute respiratory syndrome virus 2 (SARS-CoV-2) causing the related coronavirus disease (COVID-19) has been spreading worldwide from Wuhan [6] and, in Italy, the first case of SARS-CoV-2 was detected in the late February 2020 [7]. At the end of January 2020, the World Health Organization (WHO) officially declared the COVID-19 epidemic as a public health emergency [8].

Since the spread has already taken on pandemic proportions, affecting more than 100 countries, new issues have been posed in terms of COVID-19 diagnosis and early characterization of clinical features in order to interrupt the transmission chain of the virus. 

In this setting, the rapid identification of paucisymptomatic subjects represents a great concern in terms of public health and control of viral transmission. Recently, the COVID-19 epidemic in Europe and, in particular in Italy, has highlighted that the total or partial loss of smell and taste in SARS-CoV-2 infected patients represents an important characteristic of this disease [9,10,11].

Since increasing evidence of olfactory or gustatory dysfunction as potential early symptoms of COVID-19 infection have been reported, the Centers for Disease Control and Prevention (CDC) recently added “new loss of taste or smell” to its list of symptoms that can normally be diagnosed between 2 and 14 days after exposure to SARS-CoV-2 virus [12].

Olfactory and taste disorders (OTDs) have already been reported in patients with documented HCoVs infection: in 2006 a case of anosmia persisting more than two years in a woman with SARS was reported for the first time [13] and in 2007 Suzuki et al. identified coronavirus in the nasal discharge of patients with postviral olfactory dysfunction [14]. However, it has been observed in very few cases, representing a rare occurrence. Moreover, in COVID-19 patients, ageusia and anosmia are often not associated with nasal obstruction or other rhinitis symptoms, despite Suzuki et al. proving only for rhinovirus and olfactory dysfunction through mechanisms other than nasal obstruction [14].

Although the mechanisms underlying these symptoms are not fully elucidated, evidence of viral-induced degeneration of the olfactory mucosa have been associated with different upper respiratory viral infection [15] and a transneuronal penetration of SARS-CoV through the olfactory bulb in mice model [16]. Moreover, the wide expression of angiotensin-converting enzyme 2 receptor on the epithelial cells of the oral mucosa may act as a door for SARS-CoV-2 to penetrate into the cells and spread to the upper airways [17].

Focusing on the diagnosis of SARS-CoV-2 infection, the identification of positive infected subjects is normally based on molecular techniques, including RT-PCR for the detection of viral RNA. Recently, the urgent need of methods able to characterize SARS-CoV-2 specific serological response has emerged, since the application of serological assays in specific population-setting may represent a useful tool for understanding the virus spread and the relative seroprevalence. In detail, quantitative assays can give a result in terms of concentration of SARS-CoV-2 IgG (AU/mL), allowing the evaluation of antibody kinetics during the course of infection.

To date, the relationship between OTDs in COVID-19 positive patients and molecular and serological features of SARS-CoV-2 infection has not been fully investigated.

In this setting, the main objective of this study was to better clarify the relationship between COVID-19 and olfactory and taste dysfunctions (anosmia or hyposmia and/or ageusia or hypogeusia), as well as to investigate the virologic and serological features of COVID-19 infection in a cohort of patients reporting hyposmia and hypogeusia. Moreover, we described the kinetics of SARS-CoV-2 RNA and SARS-CoV-2 IgG by using molecular and serological quantitative assays.

The study was performed in agreement with the Helsinki declaration and approved by the Ethical Committee of IRCCS Policlinico San Matteo (Protocol Number: 20200041154) and all the patients gave their written informed consent.

## 2. Patients and Methods

### 2.1. Demographic and Clinical Characteristics of Enrolled Patients Reporting Olfactory and Taste Disorders

A total of 69 subjects (median age 43, range 19-63 years) reporting new-onset OTDs was prospectively enrolled from 25 March to 11 April 2020 and monitored for at least two weeks. Of them, 40 (58%) were females and 29 (42%) were males. We recorded clinical history to exclude concurrent sinonasal disease or surgery, a nasal decongestant or cocaine abuse, neuropsychiatric disorders and head injury. Moreover, the day of the symptoms’ onset was recorded in all the subjects. We performed only anterior rhinoscopy evaluation because endoscopy was not allowed in this type of patient. None of the patients in the study group had pathological findings in the ENT evaluation. Moreover, nasal swabs (NS) and serum samples were prospectively collected. 

### 2.2. OTD Severity was Defined According to Hyposmia Rating Scale (HRS) and Chemotherapy Induced Taste Alteration Scale (CiTAS)

A questionnaire based on the Hyposmia Rating Scale (HRS) [18] and on the Chemotherapy Induced Taste Alteration Scale (CiTAS) [19] was administered at each control to grade hyposmia and hypogeusia.

Olfactory dysfunction was graded with a VAS from 0 (worst possible) to 10 (best possible) and with the validated and self-administered HRS. HRS has the strongest correlation with the Sniffin’ Sticks Test, which is simple to administer and has good clinimetric properties. HRS consists of six questions referring to the frequency with which certain odors were perceived and ranges from 6 (best possible olfactory function) to 30 (worst olfactory function).

Taste dysfunction was graded, as olfaction, with a 0 to 10 VAS and with a questionnaire based on the CiTAS.

CiTAS is an 18-items scale that assesses specific taste alterations, symptoms of discomfort and their impact on patient nutrition during and after chemotherapy. It is a valid and reliable measurement of taste dysfunction and since the lack of a validated and specific questionnaire we chose to modify CiTAS in order obtain an eight questions survey form on quantitative changes in taste perception answered with a 5-point Likert scale, ranging from 8 (best possible taste function) to 40 (worst possible).

### 2.3. Sequential Samples’ Collection and Molecular Laboratory Testing

Sequential NS were collected at the time of enrolment (T0) and after one (T7) and two weeks (T14) and analyzed for detection and quantification of SARS-CoV-2 RNA. In detail, respiratory samples from the upper respiratory tract (FLOQSwabs™, Copan Italia, Brescia, Italy) were collected in enrolled patients at the time of enrolment and at follow-up time points. Total nucleic acids (DNA/RNA) were extracted from 400 µl of UTM™ using the QIAsymphony® instrument with QIAsymphony® DSP Virus/Pathogen Midi Kit (Complex 400 protocol) according to the manufacturer’s instructions (QIAGEN, Qiagen, Hilden, Germany). Specific real-time RT-PCRs targeting RNA-dependent RNA polymerase and E genes were used to detect the presence of SARS-CoV-2 according to the WHO guidelines [20] and protocol reported from Corman and colleagues [21].

### 2.4. Serum Samples Collection and Serological Laboratory Testings

In parallel, sequential serum samples collected at the same time points of NS were analyzed using chemiluminescent assay (Liason SARS-CoV-2 S1/S2 IgG, Diasorin, Saluggia, Italy) for the quantitative characterization of SARS-CoV-2 anti-S1 and anti-S2 IgG antibodies, according to manufacturer’s instructions. Results were given as AU/mL and a cut-off of 15 AU/mL was considered for the definition of positive samples. Results ranging from 12 and 15 AU/mL were considered borderline while IgG titer less than 12 AU/mL was given as a negative result.

### 2.5. Statistical Analysis

Descriptive data were reported as absolute and relative frequencies, median and interquartile range (IQR) based on the type of the variable distribution. In detail, the frequencies of COVID-19 positive patients was reported. Analysis of COVID-19 subjects positive for SARS-CoV-2 RNA and SARS-CoV-2 IgG was reported. Mann–Whitney test was used for quantitative variables to perform a comparison between two groups. Moreover, the ANOVA test was used in order to compare more than two groups of data (HSR and CiTAS severity at time T0, T7 and T14). All tests were two-tailed and *p*  <  0.05 was considered statistically significant. All the analyses were performed using GraphPad Prism 5 (GraphPad Software, La Jolla, CA, USA).

## 3. Results

### 3.1. Analysis of Demographic and Clinical Characteristics of 69 OTDs Patients and SARS-CoV-2 Prevalence

Among the 69 enrolled patients, 52 (75.4%) were diagnosed as COVID-19 positive, being SARS-CoV-2 RNA and/or SARS-CoV-2 IgG positive, while 17 (24.6%) were consistently negative and excluded from the subsequent analyses. Among the 52 COVID-19 positive patients, 34 (65.4%) were positive for SARS-CoV-2 RNA while the other 18 (34.6%) tested negative. In addition, 39 patients developed a detectable SARS-CoV-2 IgG titer. Of them, 22 (56.4%) were also SARS-CoV-2 RNA positive, while 17 (43.6%) tested positive only by serology.

All the demographic and clinical characteristics of the COVID-19 positive patients, including smoking, comorbidities and specific symptoms were considered. The large part of enrolled subjects never smoked (69.2%) and did not show comorbidities (73.1%); seasonal influenza vaccination was reported only in 13.5% of the individuals while none traveled in risky areas for COVID-19. The great majority of subjects reported combined perceptive disorders (92.3%), while less than 8% of COVID-19 positive subjects reported only olfactory disorder. All the details have been reported in Table 1.

**Figure 1:**
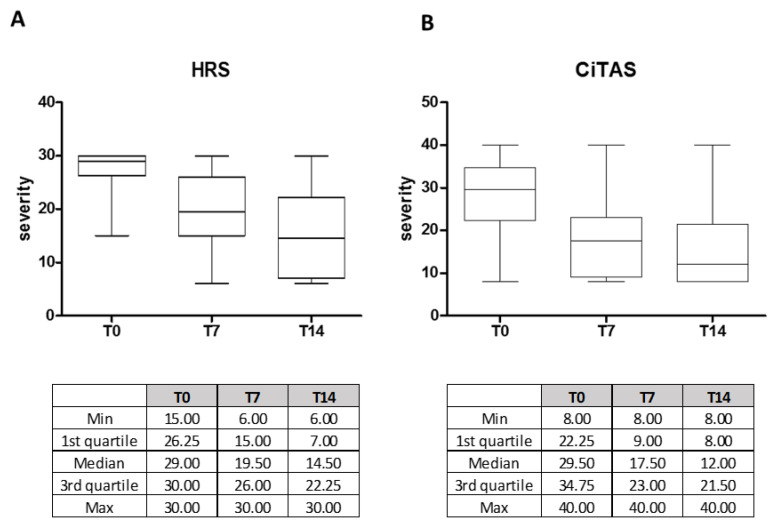
Olfactory and taste disorder severity at T0, T7 and T14 scored according to HSR (**A**) and a questionnaire based on CiTAS (**B**) in COVID-19 positive patients. All the relevant statistics values are reported in the corresponding tables.

OTDs severity, evaluated at T0, T7 and T14 was described in Figure 1. In detail, the median of HSR severity at the time of enrolment was 29.00 (IQR 26.25–30.00) and subsequently decreased to 19.50 (IQR 15.00–26.00) at time T7 and to 14.50 (IQR 7.00–22.25) at time T14 (*p* < 0.0001).

Similarly, a decrease in terms of severity of CiTAS was observed during the follow-up, ranging from a median of 29.50 (IQR 22.25–34.75) at time T0 to 17.50 (IQR 9.00–23.00) at time T7 and to 12.00 (IQR 8.00–21.50) at time T14 (*p* < 0.0001).

### 3.2. SARS-CoV-2 RNA and SARS-CoV-2 IgG Trend in 52 COVID-19 Patients Analyzed at Different Time Points

Data about the symptoms’ onset were available for all the 52 COVID-19 positive patients. The median time from symptoms’ onset to enrolment was 12 days (IQR 7–17 days). Interestingly, we observed that the median number of days ranging from symptoms’ onset and enrolment was significantly lower in patients with positive SARS-CoV-2 RNA in NS (median 10.0 days IQR 5.75–14) than those who tested negative for SARS-CoV-2 RNA in NS (median 15.5 days IQR 12.75–24.75; *p* = 0.0024), suggesting that symptoms might last longer than SARS-CoV-2 replication.

**Figure 2:**
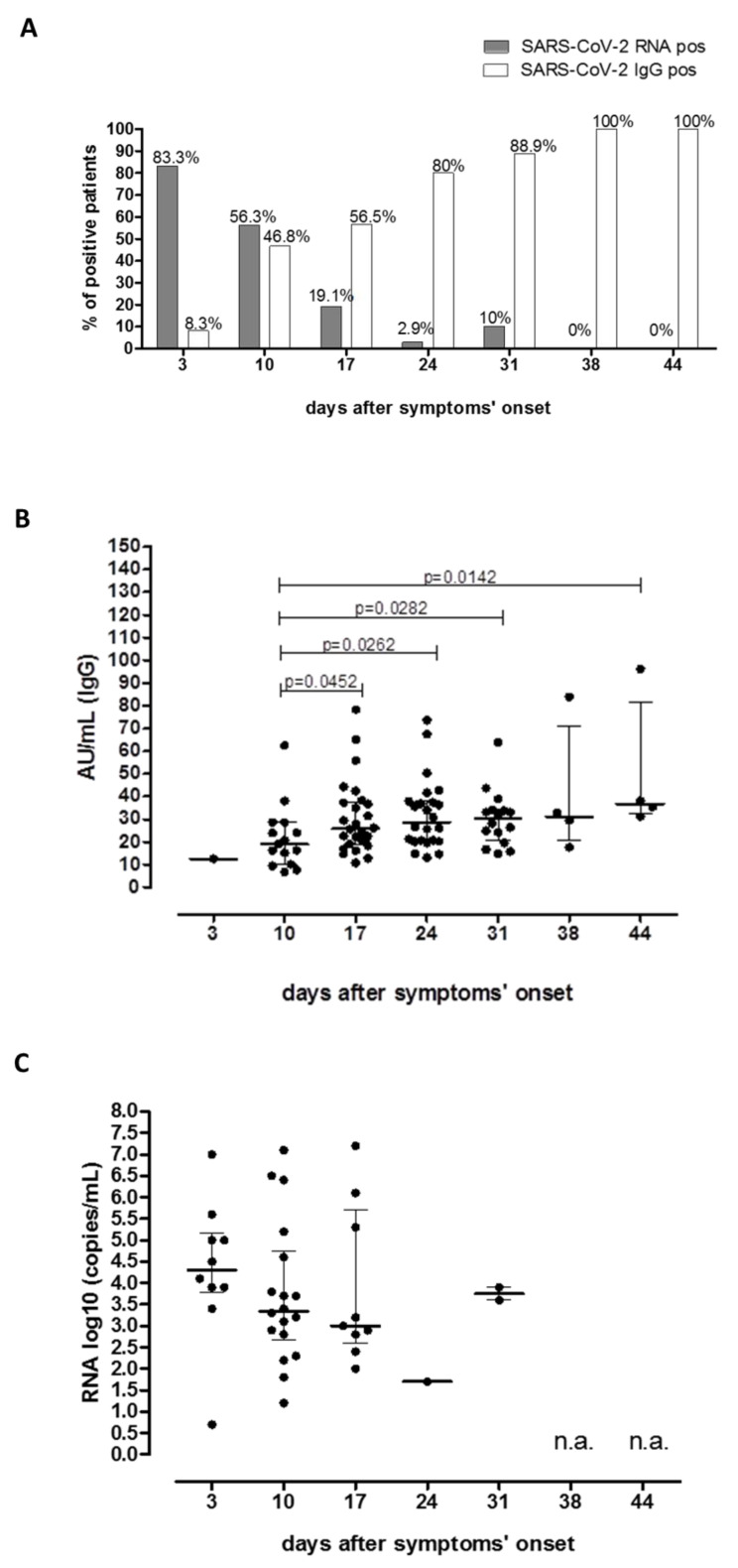
Prevalence and kinetics of SARS-CoV-2 RNA and SARS-CoV-2 IgG positive patients in a cohort of 52 COVID-19 positive anosmic and aguesic patients since symptoms’ onset. Prevalence of SARS-CoV-2 RNA positive patients (dark gray bars) and SARS-CoV-2 IgG positive patients (white bars) was evaluated according to days after symptoms’ onset. Percentage of positive patients was given for each bar (**A**). Median SARS-CoV-2 IgG titer (**B**) and SARS-CoV-2 SARS-CoV-2 RNA (**C**) were evaluated during the follow-up. *P*-values for significant comparison were given for each graph. n.a: not available (not positive results reported).

Distribution of SARS-CoV-2 seroprevalence as well as the detectability of SARS-CoV-2 RNA were evaluated over time, with respect to symptoms’ onset. Overall, we observed that the rate of patients positive for SARS-CoV-2 RNA rapidly decreased from 83.3% (10/12) at three days from symptoms’ onset to 2.9% (1/35) at 24 days from symptoms’ onset. On the other hand, SARS-CoV-2 RNA was still positive in 10% (2/20) of the patients at day 31. Interestingly, no patient tested positive for SARS-CoV-2 RNA in NS at 38 and 44 days after symptoms’ onset. Conversely, positive patients for SARS-CoV-2 IgG increased from 8.3% (1/12) at three days from symptoms’ onset to 100% (4/4) at day 38 and 44 after symptoms’ onset (Figure 2A).

We also reported that median level of IgG titer increased significantly during the period of follow-up since day 10 (median 19.10 AU/mL IQR 10.20–28.60 AU/mL) to day 44 (median 36.9 AU/mL IQR 32.3–81.7 AU/mL; *p* = 0.0142; Figure 2B). 

At the same time, a decreasing trend of median log_10_ copies/mL of SARS-CoV-2 RNA from Day 3 since symptoms’ onset to day 24 was reported, although no statistical significance was observed (Figure 2C). 

## 4. Discussion

The newly emerged COVID-19 is spreading worldwide, threatening the world. According to the earlier findings, the main common symptoms are fever, cough, breath dysfunction and sore throat. 

Several reports described an increased incidence of COVID-19 infection in males, who showed normally severe symptoms [22,23]. In our setting, the prevalence of COVID-19 positive males was approximately 45%. From a clinical point of view, the severity of the disease may be related to the immune system impairment being the cytokine storm responsible for lung tissue damage and immunopathogenesis of COVID -19 infection. The cytokine storm represents a rapid acute increase in circulating levels of different proinflammatory cytokines including IL-6, IL-1 and TNF- α [24].

In this setting, therapeutic approaches that are able to suppress inflammatory cytokines production have been considered for the treatment of severe COVID-19 disease [25,26].

Although the prevalence of SARS-CoV-2 in pediatric subjects is normally low and infection is mild or asymptomatic [27,28], recently a possible association between Kawasaki-like disease and SARS-CoV-2 spread has emerged [29,30,31,32]. As in COVID-19, Kawasaki disease and its similar forms are mainly mediated by proinflammatory cytokines and, even if the cause of the disease is still unknown, evidence suggests that an infectious agent triggers a cascade that causes the illness [33]. Based on this evidence, the blocking of proinflammatory cytokines may represent an effective therapeutic strategy [27].

To date, therapeutic approaches aimed to reduce the COVID-19 inflammatory phase have been proposed, including tocilizumab and ruxolitinib [34]. On the other side, the use of immunosuppressants like corticosteroid remains controversial [35,36]. The most promising one is represented by dexamethasone, a corticosteroid used in a wide range of conditions for its anti-inflammatory and immunosuppressive effects [37].

Despite SARS-CoV-2 being responsible for severe diseases, it was also found that a large part of SARS-CoV-2 infected subjects is mild symptomatic or completely asymptomatic. For this reason, the rapid identification of asymptomatic and paucisymptomatic COVID-19 positive patients represents the main issue in order to limit the transmission of SARS-CoV-2 and its spread. 

Interestingly, olfactory and gustatory dysfunctions have been frequently reported as COVID19-related symptoms [9,10,38], thus suggesting that impairment of smell and taste may represent an early feature for the identification and then the isolation of COVID-19 positive subjects and, sometimes, the only one. In other reports, it was observed that about one-third of COVID-19 positive subjects complained of olfactory or gustatory dysfunctions [10]. 

Although olfactory loss may represent a common feature in the setting of upper respiratory infections, the underlined pathogenic mechanisms responsible for COVID-19-mediated olfactory and/or gustatory disturbances has not yet been definitively identified [39].

Several studies aimed to characterize the prevalence of anosmia and ageusia in COVID-19 infected people [10,38,40,41,42,43] in many cases OTDs are referred as a first or only symptom in COVID-19 patients [27] and it was reported that self-reported olfactory and taste disorders could have a specificity higher than 95% specificity as a screening criterion for COVID-19 [44].

Interestingly, based on the results obtained from our study, about 25% of patients with OTDs were negative for both SARS-CoV-2 RNA and SARS-CoV-2 IgG and they were then considered COVID-19 negative, while the other 75% showed a proven COVID-19 infection. In other studies, the prevalence of COVID-19 infection in a cohort of subjects with olfactory disorders reached 87.5% [45].

In addition, about one-third of COVID-19 positive subjects in our cohort were detected only by serological assays, testing always negative for SARS-CoV-2 RNA. This was probably due to the fact that there was great heterogeneity in our cohort in terms of symptoms’ onset. Indeed, we observed that those subjects enrolled at early symptoms’ onset were positive for SARS-CoV-2 RNA; otherwise, the median range between symptoms’ onset and enrolment was significantly higher in patients with negative SARS-CoV-2 RNA. Accordingly, Zhao et al. reported that the overall RNA positive rate was lower than 70% in patients in the first-week post symptom onset and fell to 50% in the next week [46]. Similarly, also in our cohort of subjects, the detectability of SARS-CoV-2 RNA decreased dramatically from about 83% to less than 3% in a few weeks after symptoms’ onset.

Based on these results, the diagnostic potential of serological assays is crucial for the definition of seroprevalence of SARS-CoV-2 in specific setting populations. We also previously reported that in the cohort settings of asymptomatic blood donors referred to one of the most critical areas of Northern Italy (Lodi Red Zone), the seroprevalence of SARS-CoV-2, analyzed by microneutralization assay, was approximately 23%. Interestingly in this setting, the prevalence of SARS-CoV-2 RNA was less than 5%, supporting the diagnostic potential of the serological approach in the definition of SARS-CoV-2 infected subjects [47].

Despite the prevalence of OTDs seems to be higher in COVID-19 patients [43], anosmia and ageusia have already been reported as part of the clinical manifestation in other viral infections [14,48,49,50]. Indeed, it has been previously observed that about a quarter of patients could develop anosmia as a result of an upper respiratory infection or cold, which could be related to neuroepithelial dysfunction [51]. Moreover, Pellegrino et al. described that the prevalence of OTDs was approximately about 60% in common cold settings [52]. According to this evidence, we reported that a quarter of subjects in our cohort was negative for COVID-19, supporting the concept that a differential diagnosis with other respiratory viruses or respiratory syndromes could be useful.

In this study, we also contribute to the definition of a serological and molecular pattern of SARS-CoV-2 in COVID-19 infected subjects, analyzing the kinetics of both SARS-CoV-2 RNA and SARS-CoV-2 IgG according to symptoms’ onset.

By monitoring COVID-19 positive patients for serological features, we reported that less than 10% of patients were positive for SARS-CoV-2 IgG titer at three days after symptoms’ onset, while the seroprevalence reached the 80% after approximately three weeks after the symptoms’ onset. In line with our results, it was reported that the antibody levels increased rapidly starting at six days postonset with a parallel decline in viral load [53].

Overall, we noticed that a non-negligible portion of patients (11/52; 21.2%) reported only OTDs as suggestive symptoms for COVID-19 clinical diagnosis, according to previous studies [9]. In our cohort of patients, although OTDs were severe at the onset, we observed a trend of progressive improvement in the majority of cases. Indeed we observed a progressive significant reduction in terms of HSR and CiTAS scales between the time of enrolment and after 14 days from the enrolment.

In conclusion, despite OTDs might represent an important feature for early identification of COVID-19 positive patients, and in some cases the only one, differential diagnosis with other respiratory diseases is necessary. On the other hand, only relying on nasal swab RT-PCR might lead to a false negative diagnosis, especially in patients presenting several days after symptoms onset, when the proportion of cases with replicative infection in the nasal cavity appears to drop. In these cases, serological assays could be crucial for the identification of COVID-19-positive cases and strict contact tracing.

Additional analyses in larger sample settings might be useful for a better definition of SARS-CoV-2 RNA and IgG kinetics. Furthermore, the characterization of long-term persistence and, eventually, of the potential protective role of SARS-CoV-2 antibodies in naturally infected subjects represent an interesting field of research.

## Figures and Tables

**Table 1:** Clinical features of COVID-19 positive patients with olfactory and taste disorders.

Patients	N = 52	
Age (median range)	41 (19–58)	
Female	29	55.8%
		
**Smoking**		
Never	36	69.2%
Previous smoker	7	13.5%
Active smoker	9	17.3%
		
**Comorbidities**		
None	38	73.1%
Hypertension	4	7.7%
Cardiovascular	1	1.9%
Neoplastic	1 (parotid adenoma)	1.9%
Hormonal	1 (Hashimoto’s disease)	1.9%
Other (diverticulosis, psoriasis, etc.)	7	13.5%
**Allergy**	10	19.2%
		
**Sleep-related breathing disorders**		
Snoring	5	9.6%
None	47	90.4%
		
**Seasonal influenza vaccination**	7	13.5%
**Travel in risky areas for COVID-19**	0	0
**Contact with COVID-19 patients**	18	34.6%
		
**Sinonasal sign and symptoms**		
Nasal obstruction	12	23.1%
Rhinorrhea	12	23.1%
Epistaxis	0	0
Cacosmia	3	5.8%
		
**Olfactory disorder only**		
Anosmia	4	7.7%
Hyposmia	0	0
**Taste disorder only**	0	0
		
**Combined perceptive disorder**		
Anosmia and ageusia	38	73.1%
Anosmia and hypogeusia	5	9.6%
Hyposmia and ageusia	0	0
Hyposmia and hypogeusia	5	9.6%
		
**COVID-19 symptoms**		
OTDs only	11	21.2%
Contemporary onset of OTDs and flu-like symptoms (fever, cough, asthenia, diarrhea)	11	21.2%
OTDs before flu-like symptoms	3	5.8%
OTDs after flu-like symptoms	27	51.8%

## References

[1] Su S., Wong G., Shi W., Liu J., Lai A.C.K., Zhou J., Liu W., Bi Y., Gao G.F. (2016). Epidemiology, Genetic Recombination, and Pathogenesis of Coronaviruses. Trends Microbiol..

[2] Zhong N.S., Zheng B.J., Li Poe Y.M., Xie Z.H., Chan K.H., Li P.H., Tan S.Y., Chang Q., Xie J.P., Liu X.Q. (2020). Epidemiology and cause of severe acute respiratory syndrome (SARS) in Guangdong, People’s Republic of China, in February, 2003. Lancet.

[3] Zaki A.M., van Boheemen S., Bestebroer T.M., Osterhaus A.D., Fouchier R.A. (2012). Isolation of a novel coronavirus from a man with pneumonia in Saudi Arabia. N. Engl. J. Med..

[4] Wu D., Wu T., Liu Q., Yang Z. (2020). The SARS-CoV-2 Outbreak: What We Know. Int. J. Infect. Dis..

[5] Cui J., Li F., Shi Z.L. (2019). Origin and evolution of pathogenic coronaviruses. Nat. Rev. Microbiol..

[6] Zhu N., Zhang D., Wang W., Li X., Yang B., Song J., Zhao X., Huang B., Shi W., Lu R. (2020). China Novel Coronavirus Investigating and Research Team. A Novel Coronavirus from Patients with Pneumonia in China, 2019. N. Engl. J. Med..

[7] Livingston E., Bucher K. (2020). Coronavirus Disease 2019 (COVID-19) in Italy. JAMA.

[8] Guo Y.R., Cao Q.D., Hong Z.S., Tan Y.Y., Chen S.D., Jin H.J., Tan K.S., Wang D.Y., Yan Y. (2020). The Origin, Transmission and Clinical Therapies on Coronavirus Disease 2019 (COVID-19) Outbreak—An Update on the Status. Mil. Med. Res..

[9] Vaira L.A., Salzano G., Deiana G., De Riu G. (2020). Anosmia and ageusia: Common findings in COVID-19 patients. Laryngoscope.

[10] Giacomelli A., Pezzati L., Conti F., Bernacchia D., Siano M., Oreni L., Rusconi S., Gervasoni C., Ridolfo A.L., Rizzardini G. (2020). Self-reported olfactory and taste disorders in SARS-CoV-2 patients: A cross-sectional study. Clin. Infect. Dis..

[11] Spinato G., Fabbris C., Polesel J., Cazzador D., Borsetto D., Hopkins C., Boscolo-Rizzo P. (2020). Alterations in smell or taste in mildly symptomatic outpatients with SARS-CoV-2 infection. JAMA.

[12] Centers for Disease Control and Prevention (2020). Coronavirus Disease 2019 (COVID-19)—Symptoms. https://www.cdc.gov/coronavirus/2019-ncov/symptoms-testing/symptoms.html.

[13] Chi-Shin Hwang C.S. (2006). Olfactory Neuropathy in Severe Acute Respiratory Syndrome: Report of A Case. Acta Neurol. Taiwan.

[14] Suzuki M., Saito K., Min W.P., Vladau C., Toida K., Itoh H., Murakami S. (2007). Identification of Viruses in Patients with Postviral Olfactory Dysfunction. Laryngoscope.

[15] Yamagishi M., Fujiwara M., Nakamura H. (1994). Olfactory mucosal findings and clinical course in patients with olfactory disorders following upper respiratory viral infection. Rhinology.

[16] Netland J., Meyerholz D.K., Moore S., Cassell M., Perlman S. (2008). Severe acute respiratory syndrome coronavirus infection causes neuronal death in the absence of encephalitis in mice transgenic for human ACE2. J. Virol..

[17] Xu H., Zhong L., Deng J., Peng J., Dan H., Zeng X., Li T., Chen Q. (2020). High expression of ACE2 receptor of 2019-nCoV on the epithelial cells of oral mucosa. Int. J. Oral. Sci..

[18] Millar Vernetti P., Perez Lloret S., Rossi M., Cerquetti D., Merello M. (2012). Validation of a new scale to assess olfactory dysfunction in patients with Parkinson’s disease. Parkinsonism Relat. Disord..

[19] Campagna S., Gonella S., Stuardi M., Sperlinga R., Cerponi M., Olivero M., Giuliano P.L., Marchese R., Carnovali E., Pedersini R. (2016). Validazione italiana della Chemotherapy induced Taste Alteration Scale (CiTAS) [Italian validation of the Chemotherapy Induced Taste Alteration Scale]. Assist. Inferm. Ric..

[20] WHO Novel Coronavirus—China. http://wwwwhoint/csr/don/12-january-2020-novel-coronavirus-china/en/.

[21] Corman V.M., Landt O., Kaiser M., Molenkamp R., Meijer A., Chu D.K., Bleicker T., Brünink S., Schneider J., Schmidt M.L. (2020). Detection of 2019 novel coronavirus (2019-nCoV) by real-time RT-PCR. EuroSurveill.

[22] Conti P., Younes a. (2020). Coronavirus COV-19/SARS-CoV-2 Affects Women Less Than Men: Clinical Response to Viral Infection. J. Biol. Regul. Homeost. Agents.

[23] Falahi S., Azra Kenarkoohi A. (2020). Sex and Gender Differences in the Outcome of Patients With COVID-19. J. Med. Virol..

[24] Ragab D., Salah Eldin H.S., Taeimah M., Khattab R., Salem R. (2020). The COVID-19 Cytokine Storm; What We Know So Far. Front. Immunol..

[25] Conti P., Gallenga C.E., Tetè G., Caraffa A., Ronconi G., Younes A., Toniato E., Ross R., Kritas S.K. (2020). How to reduce the likelihood of coronavirus-19 (CoV-19 or SARS-CoV-2) infection and lung inflammation mediated by IL-1. J. Biol. Regul. Homeost. Agents.

[26] Conti P., Ronconi G., Caraffa A., Gallenga C., Ross R., Frydas I., Kritas S. (2020). Induction of Pro-Inflammatory Cytokines (IL-1 and IL-6) and Lung Inflammation by Coronavirus-19 (COVI-19 or SARS-CoV-2): Anti-Inflammatory Strategies. J. Biol. Regul. Homeost. Agents.

[27] Rovida F., Cereda D., Novati S., Licari A., Triarico A., Marseglia G.L., Bruno R., Baldanti F. (2020). San Matteo Pavia COVID-19 Task Force Low Risk for SARS-CoV2 Symptomatic Infection and Early Complications in Paediatric Patients During the Ongoing CoVID19 Epidemics in Lombardy. Clin. Microbiol. Infect..

[28] Ong J.S.M., Tosoni A., Kim Y., Kissoon N., Murthy S. (2020). Coronavirus Disease 2019 in Critically Ill Children: A Narrative Review of the Literature. Pediatr. Crit. Care Med..

[29] Verdoni L., Mazza A., Gervasoni A., Martelli L., Ruggeri M., Ciuffreda M., Bonanomi E., D’Antiga L. (2020). An Outbreak of Severe Kawasaki-like Disease at the Italian Epicentre of the SARS-CoV-2 Epidemic: An Observational Cohort Study. Lancet.

[30] Riphagen S., Gomez X., Gonzalez-Martinez C., Wilkinson N., Theocharis P. (2020). Hyperinflammatory shock in children during COVID-19 pandemic. Lancet.

[31] Viner R.M., Whittaker E. (2020). Kawasaki-like disease: Emerging complication during the COVID-19 pandemic. Lancet.

[32] Ronconi G., Teté G., Kritas S.K., Gallenga C.E., Caraffa A., Ross R., Conti P. (2020). SARS-CoV-2, which induces COVID-19, causes kawasaki-like disease in children: Role of pro-inflammatory and anti-inflammatory cytokines. J. Biol. Regul. Homeost. Agents.

[33] Rowley A.H. (2018). Is Kawasaki Disease an Infectious Disorder?. Int. J. Rheum. Dis..

[34] Magro G. (2020). COVID-19: Review on Latest Available Drugs and Therapies Against SARS-CoV-2. Coagulation and Inflammation Cross-Talking. Virus Res..

[35] Theoharides T.C., Conti P. (2020). Dexamethasone for COVID-19? Not so fast. J. Biol. Regul. Homeost. Agents..

[36] Russell C.D., Millar J.E., Baillie J.K. (2020). Clinical evidence does not support corticosteroid treatment for 2019-nCoV lung injury. Lancet.

[37] (2020). RECOVERY Trial. https://www.recoverytrial.net/results.

[38] Lechien J.R., Chiesa-Estomba C.M., De Siati D.R., Horoi M., Le Bon S.D., Rodriguez A., Dequanter D., Blecic S., El Afia F., Distinguin L. (2020). Olfactory and gustatory dysfunctions as a clinical presentation of mild-to-moderate forms of the coronavirus disease (COVID-19): A multicenter European study. Eur. Arch. Otorhinolaryngol..

[39] Soler Z.M., Patel Z.M., Turner J.H., Holbrook E.H. (2020). A primer on viral-associated olfactory loss in the era of COVID-19. Int. Forum Allergy Rhinol..

[40] Vaira L.A., Hopkins C., Salzano G., Petrocelli M., Melis A., Cucurullo M., Ferrari M., Gagliardini L., Pipolo C., Deiana G. (2020). Olfactory and gustatory function impairment in COVID-19 patients: Italian objective multicenter-study. Head Neck.

[41] Tong J.Y., Wong A., Zhu D., Fastenberg J.H., Tham T. (2020). The Prevalence of Olfactory and Gustatory Dysfunction in COVID-19 Patients: A Systematic Review and Meta-analysis. Otolaryngol. Head Neck Surg..

[42] Beltrán-Corbellini Á., Chico-García J.L., Martínez-Poles J., Rodríguez-Jorge F., Alonso-Cánovas A. (2020). Acute-onset smell and taste disorders in the context of Covid-19: A pilot multicenter PCR-based case-control study. Eur. J. Neurol..

[43] Xydakis M.S., Dehgani-Mobaraki P., Holbrook E.H., Geisthoff U.W., Bauer C., Hautefort C., Herman P., Manley G.T., Lyon D.M., Hopkins C. (2020). Smell and taste dysfunction in patients with COVID-19. Lancet Infect. Dis..

[44] Wee L.E., Chan Y.F.Z., Teo N.W.Y., Cherng B.P.Z., Thien S.T., Won H.M., Wijaya L., Toh S.T., Tan T.T. (2020). The role of self-reported olfactory and gustatory dysfunction as a screening criterion for suspected COVID-19. Eur. Arch. Otorhinolaryngol..

[45] Lechien J.R., Hopkins C., Saussez S. (2020). Sniffing out the evidence; It’s now time for public health bodies recognize the link between COVID-19 and smell and taste disturbance. Rhinology.

[46] Zhao J., Yuan Q., Wang H., Liu W., Liao X., Su Y., Wang X., Yuan J., Li T., Li J. (2020). Antibody responses to SARS-CoV-2 in patients of novel coronavirus disease 2019. Clin. Infect. Dis..

[47] Percivalle E., Cambiè G., Cassaniti I., Vecchio Nepita E., Maserati R., Ferrari A., Di Martino R., Isernia P., Mojoli F., Bruno R. (2020). Prevalence of SARS-CoV-2 specific neutralising antibodies in blood donors from the Lodi Red Zone in Lombardy, Italy, as at 06 April 2020. EuroSurveill.

[48] de Haro-Licer J., Roura-Moreno J., Vizitiu A., González-Fernández A., González-Ares J.A. (2013). Long term serious olfactory loss in colds and/or flu. Acta Otorrinolaringol. Esp..

[49] Hummel T., Landis B.N., Hüttenbrink K.B. (2011). Smell and taste disorders. GMS Curr. Top. Otorhinolaryngol. Head Neck Surg..

[50] van Riel D., Verdijk R., Kuiken T. (2015). The olfactory nerve: A shortcut for influenza and other viral diseases into the central nervous system. J. Pathol..

[51] Deems D.A., Doty R.L., Settle R.G., Moore-Gillon V., Shaman P., Mester A.F., Kimmelman C.P., Brightman V.J., Snow J.B. (1991). Smell and Taste Disorders, a Study of 750 Patients From the University of Pennsylvania Smell and Taste Center. Arch. Otolaryngol. Head Neck Surg..

[52] Pellegrino R., Walliczek-Dworschak U., Winter G., Hull D., Hummel T. (2017). Investigation of chemosensitivity during and after an acute cold. Int. Forum Allergy Rhinol..

[53] Lou B., Li T.D., Zheng S.F., Su Y.Y., Li Z.Y., Liu W., Yu F., Ge S.X., Zou Q.D., Yuan Q. (2020). Serology characteristics of SARS-CoV-2 infection since exposure and post symptom onset. Eur. Respir. J..

